# Real-world outcomes of patients with renal cell carcinoma, surgically treated at regional hospitals, based on a prospective long-term survey of the pre-robotic era

**DOI:** 10.1007/s11255-023-03477-5

**Published:** 2023-02-13

**Authors:** Yoshihide Kawasaki, Hideo Saito, Naomasa Ioritani, Tatsuo Tochigi, Isamu Numata, Kenji Numahata, Fumihiko Soma, Atsushi Kyan, Shigeto Ishidoya, Shozo Ota, Takashige Namima, Kazuhiko Orikasa, Shinichi Yamashita, Koji Mitsuzuka, Yoichi Arai, Akihiro Ito

**Affiliations:** 1 Tohoku Urological Evidence-Based Medicine Study Group, Japan (Japan Community Health Care Organization Sendai Hospital, Miyagi Cancer Center, Osaki Citizen Hospital, Yamagata Prefectural Central Hospital, Hachinohe City Hospital, Shirakawa Kosei General Hospital, Sendai City Hospital, Sendai Red Cross Hospital, Tohoku Rosai Hospital, Kesennuma City Hospital, Sen-en Rifu Hospital, Iwate Prefectural Iwai Hospital, KKR Tohoku Kosai Hospital, and Tohoku University Hospital), Sendai, Japan; 2 Department of Urology, Tohoku University Graduate School of Medicine, 1-1 Seiryo-Machi, Aoba-Ku, Sendai, Miyagi 980-8574 Japan

**Keywords:** Clinical T1, Regional hospital, Renal cancer, Renal function, Radical nephrectomy, Prospective cohort

## Abstract

**Purpose:**

Renal cancer surgery is frequently performed in small regional hospitals in Japan. This study evaluated the outcomes of renal cancer surgery, comparing results from the pre-robotic surgery era with those obtained with robotic surgery.

**Methods:**

This prospective cohort study was conducted on patients who underwent renal cancer surgery between 2008 and 2013 at 14 hospitals, comprising 13 regional hospitals and a university hospital, registered in the Tohoku Urological Evidence-Based Medicine Study Group. The patients’ backgrounds; perioperative data; annual postoperative renal function; and prognostic surveys, performed over a median follow-up period of 10 years were obtained.

**Results:**

In 930 surgical cases at the 14 registered hospitals, the 10-year recurrence-free survival rates of cT1a, cT1b, cT2, and cT3 were 0.9326, 0.8501, 0.5786, and 0.5101, respectively. Meanwhile, the 10-year overall survival rates were 0.9612, 0.8662, 0.7505, and 0.7209, respectively. Long-term observation in patients with cT1 showed that vessel involvement and high tumor grade were prognostic factors for recurrence. As a noteworthy fact, radical nephrectomy was performed in 53.3% of patients with cT1a at the regional hospitals. However, even in patients with preoperative chronic kidney disease stage 3, radical nephrectomy was not a prognostic factor of renal function. This indicates that compensatory mechanisms had been working for a long time in many patients who underwent radical nephrectomies without hypertension and preoperative proteinuria, which were predictors of end-stage renal disease.

**Conclusion:**

Based on a prospective long-term survey of the pre-robotic era, our results suggested no difference of the survival outcomes between the university hospital and regional hospitals. Our study provides baseline data to evaluate the outcomes of renal cancer robotic surgery, performed at regional hospitals.

**Supplementary Information:**

The online version contains supplementary material available at 10.1007/s11255-023-03477-5.

## Introduction

Since robotic partial nephrectomies (PN) became covered by Japanese insurance in 2016, the treatment of renal cancer has been increasingly performed in tertiary medical centers, such as university hospitals, in the United States and European countries. However, until recently, in Japan, surgery on several patients with renal cancer has been performed in regional hospitals with fewer than 500 beds. Owing to the centralization of surgery for renal cancer, the risk of surgical complications has been raised among patients treated in small and medium-sized hospitals [1]. In addition, it is economically challenging to introduce robotic surgical systems into these hospitals, and systemic treatment after recurrence has become complicated, making it difficult to manage renal cancer.

Although several studies have reported on the long-term prognosis of renal cancer surgery in Japan and overseas, most of them are retrospective investigations conducted in tertiary centers. Therefore, it is difficult to conclude that the long-term outcomes of renal cancer surgery reflect the real-world results obtained from various regional hospitals in our country.

In this study, data on renal cancer surgery cases were prospectively collected from 14 regional hospitals, including Tohoku University Hospital in the north-eastern region of Japan. The real-world outcomes and long-term prognoses obtained before robotic surgery were analyzed, especial for cT1 patients. We focused on cT1 patients who underwent radical nephrectomies (RN), recruited for this study, to examine their long-term renal function after undergoing bilateral renal loss. RN and partial nephrectomy (PN) comparisons were made by assessing postoperative renal function. The comparison in oncologic outcomes was considered less important in this study because of the large number of enrolled cT1a cases with a good prognosis and the small number of cT1b cases in which PN was performed. The extent of renal functional compensation (RFC) by the preserved kidneys after RN has not been adequately studied in this population. Hence, this study will provide baseline data for comparing the results of renal cancer surgery in the robotic era in Japan.

## Materials and methods

### Study population and hospitals

**Fig. 1:**
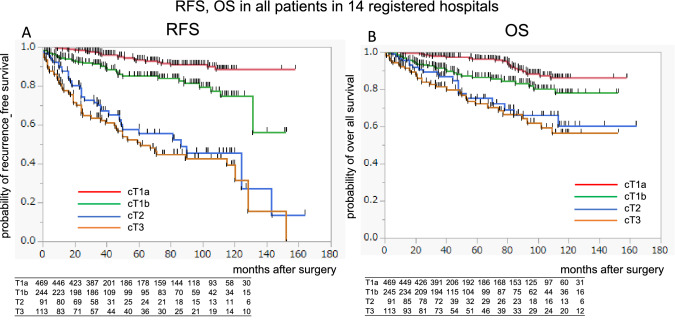
Recurrence-free survival in 14 hospitals of cT1a (red line), cT1b (blue line), cT2 (green line), and cT3 patients (orange line). **A** Overall survival of c T1a (red line), cT1b (blue line), cT2 (green line), and cT3 patients (orange line). **B**

As shown in Fig. 1, we prospectively collected the data of all patients with renal cancer who had undergone surgery from January 2008 to June 2013 at 14 institutions registered in the Tohoku Urological Evidence-Based Medicine Study Group in the north-eastern region of Japan (Supplementary figure). Data on the patients’ backgrounds, clinical stages, preoperative renal function, and pathology were investigated, and prognoses, including the postoperative renal function, was examined every year for at least 8 years (January 2008 to June 2021), after approval for extended follow-up was obtained from the Institutional Review Board (no. 2016–1-162).

Tohoku University Hospital is an educational facility in a tertiary medical center with more than 1200 beds. The 13 regional hospitals are small-scale facilities with less than 500 beds, with the exceptions of Hachinohe City Hospital and Yamagata Prefectural Central Hospital, which have more than 500 beds. Regional hospitals are staffed with three urologists (two urologists and one resident), although sometimes only two urologists are assigned, depending on residency trends. In addition, ten urologists are stationed at the University Hospital.

### Study design

In this study, patients who had undergone renal cancer surgery were prospectively investigated to analyze their long-term prognosis and postoperative renal function in the pre-robotic surgery era in Japan. The recurrence-free survival (RFS), overall survival (OS), prognostic factors, and postoperative renal function of renal cancer surgery patients in regional hospitals were compared with those in the university hospital. The indications for total and partial resection were not the same across the regional hospitals and the university hospital, as choices were made according to the skills of the urologists at the regional hospitals and the policies of the institutions. Since cT1 patients account for more than 80% of the total patients in regional hospitals, we identified cT1 patients who had undergone RN and analyzed their prognoses. All patients with a preoperative estimated glomerular filtration rate (eGFR) of greater than 15 ml/minute/1.73 m^2^ were evaluated. The compensatory renal function of patients at a regional hospital was evaluated by analyzing their postoperative renal function and the factors related to chronic kidney disease (CKD) stage 4.

### Statistical analysis

The Mann–Whitney *U* test was used to compare the patients in the subgroup analysis. The RFS and OS were evaluated using the Kaplan–Meier (KM) curve, while the prognostic factors were analyzed using univariate and multivariate analyses. The adjusted odds ratios (ORs) were calculated by performing a binomial logistic regression analysis of the clinically significant variables. Statistical significance was set at *p* < 0.05. All statistical analyses were performed using the JMP Pro software (version 15.0; SAS Institute Inc., Cary, NC, USA).

## Results

### Background of patients in regional hospitals

Table 1 shows the backgrounds of patients in regional hospitals and the university hospital. Data from 825 patients from the 13 regional hospitals and 105 from the university hospital were analyzed during the enrollment period. The patients’ median age was 64 years, and no significant differences were found in body mass index (BMI). The proportions of male patients, patients with diabetes mellitus (DM), and patients with CKD stage 3 or higher were higher in the regional hospitals than in the university hospital. Patients with clinical stages T1b to cT4 underwent RN at the university hospital, but the proportion of cT1a patients in regional hospitals was high (percentage of regional hospitals cT1a vs the university hospital: 54.6 vs 18.1%, *p* < 0001).

**Table 1:** Comparison of patient backgrounds in 13 regional hospitals and the university hospital

	Total cases (*N* = 930)	Regional hospitals (*N* = 825)	University hospital (*N* = 105)	*P*
Median Age (IQR)	64 (57, 74)	64 (56, 74)	64 (58, 74)	0.689
No. Sex (%)				
Male	646 (69.4)	588 (71.2)	58 (55.2)	< 0.0001
Median BMI (IQR)	24 (22.3 26.1)	24 (22.1, 26.3)	23 (20.8, 25.6)	0.230
No. cT sgate (%)				
cT1a	469 (50.4)	450 (54.5)	19 (18.1)	< 0.0001
cT1b	245 (26.3)	217 (26.3)	28 (26.7)	
cT2	91 (9.8)	76 (9.2)	15 (14.3)	
cT3	113 (12.2)	76 (9.2)	37 (35.2)	
cT4	12 (1.3)	6 (0.7)	6 (5.7)	
DM (%)	20.9	21.9	13.3	0.011
HT (%)	16.0	15.2	22.8	0.006
RN rate for cT1a (%)	253/469 (53.9)	249/450 (53.3)	4/19 (21.1)	< 0001
RN rate for cT1b (%)	222/245 (90.6)	195/217 (89.9)	27/28 (96.4)	0.321
Laparoscopic RN rate for cT1 (%)	323.492 (65.7)	294/461 (63.8)	29/31 (93.5)	< 0001
Median Preope. eGFR	58 (46, 76.5)	57 (45, 75.6)	63.55 (43.9, 77.6)	0.264
CKD stage > 3 (%)	465 (50.0)	419 (50.8)	46 (43.8)	< 0001
CKD stage > 4 (%)	94 (10.1)	82 (0.99)	7 (0.67)	–

### Oncologic outcomes

**Fig. 2:**
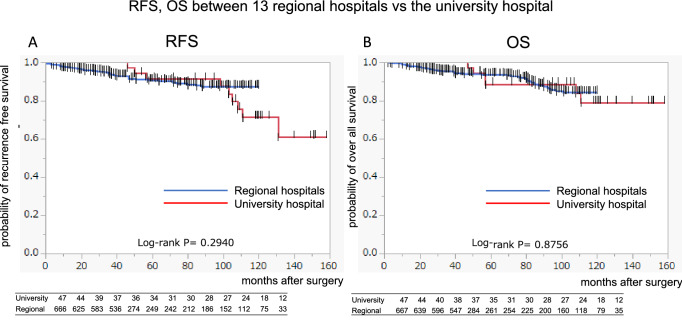
The recurrence-free survival (**A**) and overall survival (**B**) of all surgical cT1 to cT3 patients in 13 regional hospitals (blue line) and the university hospital (red line) are shown

According to the KM curves of cT1a, T1b, T2, and T3 in the 14 registered hospitals, the 10 year RFS rates were 0.932, 0.850, 0.579, and 0.510, respectively (Fig. 1A). The 10 year overall survival (OS) rates in the 14 registered hospitals were 0.961, 0.866, 0.751, and 0.721, respectively (Fig. 1B). Figure 2 shows the RFS and OS of cT1a to cT3 cases in the 13 regional hospitals and the university hospital. Although the RFS decreased in several high-risk cases in the university hospital, no significant difference was observed in terms of OS.

**Fig. 3:**
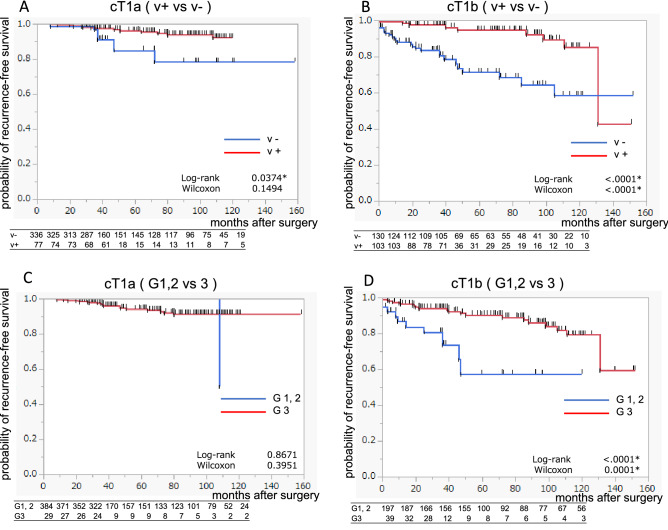
Results of the Kaplan-Meyer curve analysis of cT1 patients in 14 registered hospitals are shown. **A**−**D** show recurrence-free survival of cT1a v + vs v − patients, cT1b v + vs v − patients, cT1a G1–2 vs G3 patients, cT1b G1–2 vs G3 patients, respectively

Focusing on cT1 cases, which were predominant in all 14 of the registered hospitals, as shown in Table 2, cT1b, vessel involvement positive (v+), and G3 were independent factors contributing to the recurrence, but the facilities were not significant factors. Figure 3 shows the RFS KM curves for cT1a or cT1b patients according to the v status or tumor grade in the 14 registered hospitals. In the cT1a and cT1b cases, v+ significantly worsened the prognosis compared to vessel involvement negative (v−) (log-rank *p* = 0.0374 and < 0001, respectively). The 10-year RFS rates were 0.784 and 0.644, respectively (Fig. 3A, B). With regard to tumor grade G3 in the cT1a group, no significant difference was observed on the KM curve comparing G1,2 (Fig. 3C). However, the risk of recurrence was significantly higher in the cT1b group (log-rank *p* < 0001), and the 10-year RFS was 0.573 (Fig. 3D).

**Table 2:** Multivariate analyses of risk factors for recurrence in cT1 patients in 14 registered hospitals

Variable	Odds	95% CI	*P*
Age	4.95	1.08–24.70	0.0442
Sex			
Male	1.91	0.94–3.87	0.0695
BMI	0.23	0.03–1.57	0.1443
cT sgate			
cT1a	Ref	–	
cT1b	2.09	0.94–3.87	0.0248
Hospital			
Academic	Ref	–	
Regional	1.41	0.524–3.82	0.4896
Nephrectomy			
Radical	Ref	–	
Partial	1.31	0.60–2.83	0.4917
Tumor grade			
G 1, 2	Ref	–	
G3	2.62	1.31–5.25	0.0062
V involvement			
v –	Ref	–	
v +	2.55	1.409–4.64	0.0020

### Renal functional compensation and risk factors of high CKD stage

**Fig. 4:**
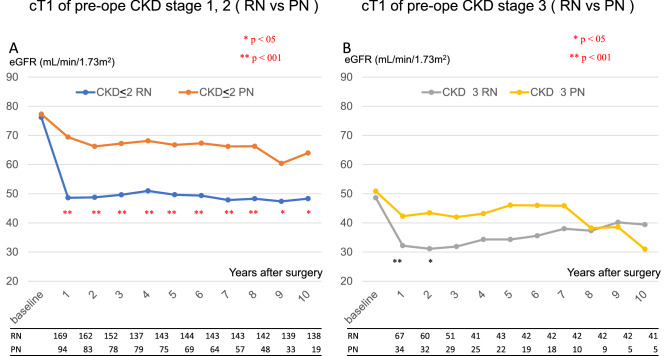
The estimated renal function within a long follow-up period by preoperative CKD stage in cT1 patients who underwent radical nephrectomy (RN) or partial nephrectomy (PN) in 14 registered hospitals is shown. **A** The blue line indicates patients with a preoperative CKD stage of 2 or below who underwent RN, while the red line indicates patients who underwent PN. **B** The grey line represents the patients with preoperative CKD stage 3 who underwent RN, while the yellow line represents the patients who underwent PN

Figure 4 shows the transition of the postoperative eGFR in cT1 patients with a preoperative CKD stage of 2 or lower (Fig. 4A) in patients with CKD stage 3 (Fig. 4B) in the 14 registered hospitals. The effect of RN on the decrease in renal function in CKD stage 3 patients was not small. However, even in patients with CKD stage 3 who had undergone RN, postoperative RFC occurred within three years after surgery, while the eGFR increased over time. Multivariate analyses demonstrated that the risk factors for CKD stage 4 in cT1 patients were preoperative proteinuria and HT (Table 3). RN and PN were not considered to be risk factors. The risk of CKD stage 4 would increase by 50 times in patients with a preoperative proteinuria of 3+ on a urine quantitative test, compared with those with a proteinuria of 2+ or lower on the urine quantitative test.

**Table 3:** Multivariate analyses of risk factors for CKD stage 4 in cT1 patients in the 14 registered hospitals

Variable	Odds	95% CI	P
Age	1.01	0.97–1.05	0.4391
Sex			
Male	1.41	0.42–0.60	0.4265
BMI	1.05	0.94–1.17	0.3568
cT sgate			
cT1a	Ref		
cT1b	1.85	0.58–5.89	0.2946
Nephrectomy			
Radical	Ref		
Partial	1.58	0.68–3.70	0.2848
Urinary protein exam			
0–2 +	Ref		
3 +	50.53	4.61–553.04	0.0013
DM			
Presence	1.44	0.62–3.32	0.3887
HT			
Presence	2.47	1.00–6.12	0.0496
CVD			
Presence	2.37	0.69–8.14	0.1692

## Discussion

Several patients had undergone renal cancer surgery in regional hospitals just before robotic surgery became widely known in Japan. The oncological outcomes of this study were comparable to those of previous studies on renal cancer surgery [2]. Of the 14 registered hospitals, 11 had fewer than 10 renal cancer surgeries per year, but the survival rates of patients with cT1 to cT3 cases were not significantly different from those of the university hospital. This could be due to the fact that the urologists in the 14 registered hospitals exchanged posts and information, and the surgical techniques were standardized to a certain extent. Occasional meetings and exchanges of information during meetings may be good for urologists in regional hospitals. However, geographical factors were reported to cause poor prognoses [1]. Compared with academic hospitals, significant differences were observed in staging at diagnosis, and the mortality rate within 30 days after surgery was extremely high (odds ratio: 4.98) at low-volume facilities, like many regional hospitals [3]. In Australia, the pT stage of renal cancer patients living in rural areas tends to be higher, and the distance to a tertiary facility influences the outcomes [1].

In the real-world data of our study, RN was performed in 53.3% of cT1a patients at regional hospitals, which was significantly higher than that performed at the university hospital. For cT1a renal cancer, PN is recommended, and RN should be selected based on the American Urological Association guidelines [4]. RN is thought to worsen the prognosis due to the decline in postoperative renal function. However, our results indicated that patients with preoperative CKD stage 3 rarely developed end-stage renal failure (< 1%, data not shown) due to the decreased renal function caused by RN. Recent studies have reported long-term functional stability and favorable survival outcomes in patients with surgically induced CKD. PN does not contribute significantly to an improvement in prognosis, compared with RN [5]. Even the well-known randomized control study EORTC30904 showed no significant difference between RN and PN in terms of long-term survival benefit in patients with an eGFR of < 30 and an eGFR of < 15 [6]. More efforts should be made to reduce the complications during PN and RN procedures, whether open or endoscopic type, which increase the risk of perioperative death and cost [7].

In all cases in our study, the recurrence rate was significantly different between cT1a and cT1b patients, and v+ and G3 for cT1b were independent factors for recurrence. Based on the National Comprehensive Cancer Network guidelines, imaging studies and renal function tests should be performed in stage I patients (cT1 without high risks) within 3–12 months after surgery and then every year for at least three years. As can be seen from the KM curve, the 10-year RFS rates of patients with cT1a with v+ and cT1b with v+ were 73 and 64%, respectively. Therefore, a long-term follow-up of approximately 10 years is required for the early detection of cancer recurrence.

It was considered that RFC after RN should start early after surgery, and that renal function would eventually stabilize within a few months. We found, however, that RFC may occur late after surgery and continue slowly even in CKD stage 3 cases. As shown in Fig. 4, RN patients showed improved renal function over a long period. The RFC after RN was greater in patients with low preoperative eGFR and larger tumors [8]. Of course, some patients experienced inadequate compensation. In our study we found that HT and the magnitude of preoperative proteinuria indicated through urine quantitative testing were risk factors for poor RFC. Reportedly, female sex, BMI, HT, and eGFR could be factors influencing RFC early after RN. The factors contributing to the late occurrence of RFC include young age, BMI, and DM [9]. Although PN has less benefit for patients with non-pre-existing CKD [10], the management of postoperative comorbidities is important, regardless of the status of RN and PN (especially in patients with HT and proteinuria), contributing to survival benefits [11, 12]. Although AUA guidelines recommend PN for patients with cT1, the survival benefit of PN was not clear in our study, partly due to the small number of severe cases of DM and HT, and partly due to the inclusion of patients in good general condition. As shown in Fig. 4B, the decrease in eGFR seven years after surgery in PN patients might be due to the fact that many of those with good renal function were lost to follow-up.

This study has some limitations. First, many of the patients withdrew from the study. Second, the preoperative baseline data, urinary albumin measurements, and background data associated with certain comorbidities were insufficient. The difference in the backgrounds of cases from the regional hospitals and from the university hospital render accurate comparison difficult. Albuminuria is an important risk factor for the exacerbation of postoperative CKD [13]. The lack of baseline data is a further limitation of this study. Third, regional bias possibly exists. However, this study reflects the actual clinical practice of renal cancer surgery based on a long-term prospective investigation of the pre-robotic surgery era in Japan.

## Conclusion

This prospective observational study of surgical cases in regional hospitals across north-eastern Japan indicated the long-term, real-world results of pre-robotic surgery for RCC. RN was performed in several cT1 patients in regional hospitals. This resulted in good prognoses, with the compensatory mechanisms maintained for a longer period to preserve renal function. These findings can be used as baseline data for determining the necessity for renal cancer surgery, which has been centralized in the robotic era.

## Supplementary Information

Below is the link to the electronic supplementary material.

Supplementary file1 The locations of the 13 regional hospitals and the university hospital in the Tohoku (north-eastern) region of Japan are shown. A total of 14 registered hospitals formed the EBM forum group. (PPTX 111 kb)

## Data Availability

Data used and/or analyzed in the current study are available upon reasonable request to the corresponding author.
