# Supplementary material for: Real-world outcomes of patients with renal cell carcinoma, surgically treated at regional hospitals, based on a prospective long-term survey of the pre-robotic era
Source: Int Urol Nephrol. 2023 Feb 13;55(4):875–82. doi: 10.1007/s11255-023-03477-5 (PMC10030418; doi:10.1007/s11255-023-03477-5)
Supplement: Supplementary file 1 — Supplementary file1 The locations of the 13 regional hospitals and the university hospital in the Tohoku (north-eastern) region of Japan are shown. A total of 14 registered hospitals formed the EBM forum group. (PPTX 111 kb) [file 11255_2023_3477_MOESM1_ESM.pptx]

## Slide 1
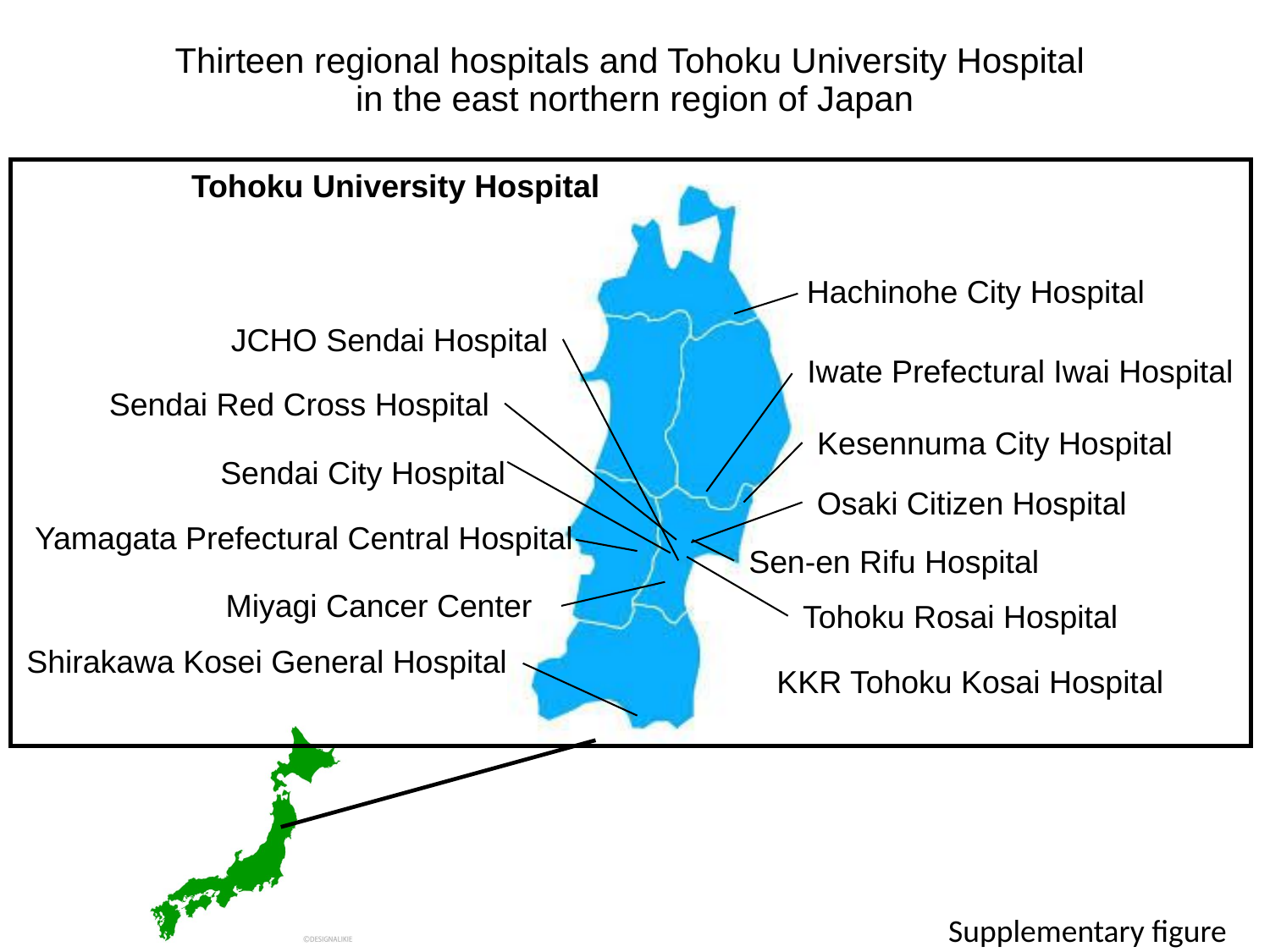

# Thirteen regional hospitals and Tohoku University Hospital in the east northern region of Japan
Hachinohe City Hospital
JCHO Sendai Hospital
Iwate Prefectural Iwai Hospital
Sendai Red Cross Hospital
Kesennuma City Hospital
Sendai City Hospital
Osaki Citizen Hospital
Yamagata Prefectural Central Hospital
Miyagi Cancer Center
Tohoku Rosai Hospital
Shirakawa Kosei General Hospital
Sen-en Rifu Hospital
Tohoku University Hospital
KKR Tohoku Kosai Hospital
Supplementary figure
